# Exploring attachment, trauma, and cannabis use in psychotic disorders: a qualitative study of patient and family perspectives

**DOI:** 10.1186/s12888-026-08022-z

**Published:** 2026-03-27

**Authors:** Samantha Carley, Robert Laprairie, Stephen Adams, G. Camelia Adams

**Affiliations:** 1 Department of Psychiatry, College of Medicine, University of Saskatchewan, Saskatoon, SK Canada; 2 College of Pharmacy and Nutrition, University of Saskatchewan, Saskatoon, SK Canada

**Keywords:** Psychosis, First-episode, Cannabis, Trauma, Attachment, Qualitative, Themes, Intervention

## Abstract

**Background:**

Psychotic disorders substantially impact individuals psychologically, occupationally, and socially. Although modifiable risk factors such as insecure attachment styles, history of psychological trauma, and substance use disorders (of which cannabis is the most frequent) are known to increase risk, it remains unclear how they may interact with each other to create pathways of vulnerability. More importantly, it is also unknown to what extent patients and their families recognize and address these risks to prevent illness, or support recovery. To address this gap, this study aimed to explore how patients and their family members understand the role of attachment, trauma, and cannabis use in the onset and recovery from psychotic disorders. Ultimately, these findings will be used to better inform interventions needed to support optimal recovery.

**Method:**

Patients and family members were recruited from the Early Psychosis Intervention Program in Saskatoon and the Schizophrenia Society of Saskatchewan. Semi-structured interviews were conducted with 17 patients experiencing first-episode psychosis and 9 family members of individuals with psychosis. Data was analyzed using reflexive thematic analysis, following Braun and Clarke’s 6-phase framework. Strategies such as reflexive journaling and triangulation across participant groups were used to ensure rigour.

**Results:**

Three major themes were generated. Theme 1 described the pre-help seeking period, marked by early confusion and misinterpretation of emerging psychotic symptoms, which often led to delays in initial help seeking, and created emergency situations. Theme 2 outlines the illness period itself, and describes participants understandings of trauma, attachment, and cannabis. While they partially understood their role in illness severity, these risks were rarely understood to have a cumulative role that create pathways of vulnerability in their illness. Finally, Theme 3 highlights recovery, which was often understood to be facilitated by family relationships, psychoeducation provided by specialized clinics, and renewed hope.

**Conclusion:**

Our findings reveal that patients and their families often have varying and inadequate understanding of the common risk factors affecting early psychosis. While the psychoeducation offered in the specialized clinic is highly valued and meaningful to recovery, it is not always accessible or sufficient to alleviate these risks. The results highlight the necessity for targeted interventions aimed at increasing knowledge translation and treatment engagement.

**Supplementary Information:**

The online version contains supplementary material available at 10.1186/s12888-026-08022-z.

## Introduction

Psychotic disorders are severe mental illnesses characterized by delusions, hallucinations, disorganized thinking and negative symptoms [1]. Although they affect only about 1% of the population [2], their impact is substantial, contributing to functional impairment, premature mortality, and significant social and economic strain [1, 3–7, 8] Despite advances in clinical care and the effectiveness of early intervention services, many individuals continue to struggle with relapse, chronicity, and barriers to recovery, including stigma and shame [9–11].

Psychotic disorders arise from a combination of genetic predispositions (e.g. inherited, unmodifiable risk factors) and environmental risks (modifiable psychosocial factors) [12–14] Three psychosocial factors are known to be especially influential in the onset and course of psychosis: insecure attachment, psychological trauma, and cannabis use [15–19] Insecure attachment, shaped early in caregiver relationships [20–22], is associated with more severe psychotic symptoms [23,24] and reduced engagement in treatment [25, 26] Psychological trauma—especially early and repeated trauma—shows strong, dose-dependent associations with psychosis, relapse, suicidality, and functional impairment [27–29], with up to 94% of individuals with psychosis reporting exposure [16] Cannabis use, particularly high-potency or frequent use, similarly increases vulnerability to psychosis and relapse, especially among youth [30–33] These concerns are amplified by global cannabis legalization, declining perceptions of harm, and increasing use among young people [34, 35] Growing evidence also indicates that these risks interact, compounding vulnerability and shaping pathways to illness and relapse [36] For example, trauma and attachment insecurity combined can heighten vulnerability to cannabis use, including use for maladaptive coping, and may exert additive effects on psychosis risk and severity [36–38] Accordingly, these key risks were selected in this study not only because of their strong empirical support and modifiable associations with psychosis, but also because together they reflect an interconnected biopsychosocial framework of vulnerability (cannabis use, psychological trauma, and attachment insecurity, respectively).

These complexities have supported the development of specialized early intervention programs using interdisciplinary teams and biopsychosocial models of care, which reduce hospitalizations, suicidality, and treatment costs [39] However, despite these advances, recovery rates remain suboptimal. In fact, when long-term outcomes are examined, specialized treatment is essentially no more effective than usual treatment [40]. This could be due to the fact that much less is known about how people with psychosis and their family members perceive, understand, or fail to understand the roles of attachment, trauma, and cannabis use in the illness.

Such insight is clinically important because understanding these risk factors may influence treatment engagement, relapse prevention long-term, recovery behaviors, and family support. Yet most existing research examines these risks in isolation, without considering how they are interpreted, connected, or navigated in everyday life.

Limited qualitative studies have begun to explore this problem by exploring how patients understand individual topics such as trauma [41, 42], cannabis use [43], or relationships [44] in relation to their illness. Some have even examined family experiences in early psychosis [45, 46] However, these studies are typically narrow in scope, as none have investigated all three risks together. Moreover, patient and family perspectives are seldom integrated, limiting a comprehensive understanding of early psychosis.

To address these gaps, this study builds on previous research by qualitatively exploring how both individuals diagnosed with early psychosis as well as their close family members perceive the role of the three risks (attachment, psychological trauma, and cannabis use) in their illness and recovery. Focusing on participants who were stable and engaged in early intervention services, we aimed to understand: how these risks were experienced; whether and how they were understood as contributing to illness, if at all; whether they were perceived as interconnected; and how they fit within broader narratives of help-seeking, treatment engagement, and recovery. This study offers a novel, integrative perspective on how patients and families make sense of these factors, and provides insights for improving awareness, mitigating risk, and supporting recovery in early psychosis care.

## Methods

### Settings and participants

Purposive sampling was used to recruit patients and family members attending specialized programs for early psychosis. Patients diagnosed with early psychosis, aged 18–35, were recruited through advertisements and referrals by staff at the Early Psychosis Intervention Program (EPIP) or the Schizophrenia Society of Saskatchewan (SSS). Patients were excluded if they had other primary psychiatric diagnoses or had severe neurological disorders or learning disabilities that could affect their ability to give informed consent or communicate in interviews. Family members were similarly recruited through clinic advertisements or referred by patients and were deemed eligible if they had a first-degree relative who met the inclusion criteria and participated in one of the two specialized programs. None of the participants recruited for this study were previously known to the first author (S. Carley), who conducted the interviews and carried out the analyses.

Recruitment was based on participant eligibility and willingness to participate, and while no formal diversity targets were set, the final sample nonetheless reflected a range of genders and personal backgrounds. Future studies could further strengthen demographic diversity through broader or national recruitment strategies.

Previous research, including a systematic review by Hennink & Kaiser (2022), indicated that anywhere from 9 to 17 participant interviews are typically needed to achieve thematic saturation in a relatively homogenous sample, which aligns with our study sample [47] Consistent with this, our sample achieved thematic saturation after (coincidently) interviewing 17 patients and 9 family members. As coding occurred concurrently with data collection, we observed that the final interviews no longer generated new concepts, but instead reinforced existing codes and patterns, indicating that the sample was sufficiently saturated to address the study aims.

### Procedure

Research ethics approval was obtained from the University of Saskatchewan Behavioural Research Ethics Board (REB #Beh-565). All procedures were conducted in accordance with REB guidelines. Prior to beginning the data collection, the interviewer (S. Carley) received training from research personnel with expertise in qualitative methods and interviewing techniques. They also completed relevant coursework in qualitative methodology as part of their preparation.

The study was advertised in the two programs through posters, and the community mental health nurses. In total, 52 individuals expressed interest in the study (40 patients and 12 family members). At this time, those who expressed interest were assigned a unique participant ID. Interested participants were contacted by an investigator either by email or phone. Those who responded and remained interested were screened for inclusion and exclusion criteria. Participants who did not progress beyond this stage were either unreachable or no longer interested due to time constraints.

**Fig. 1:**
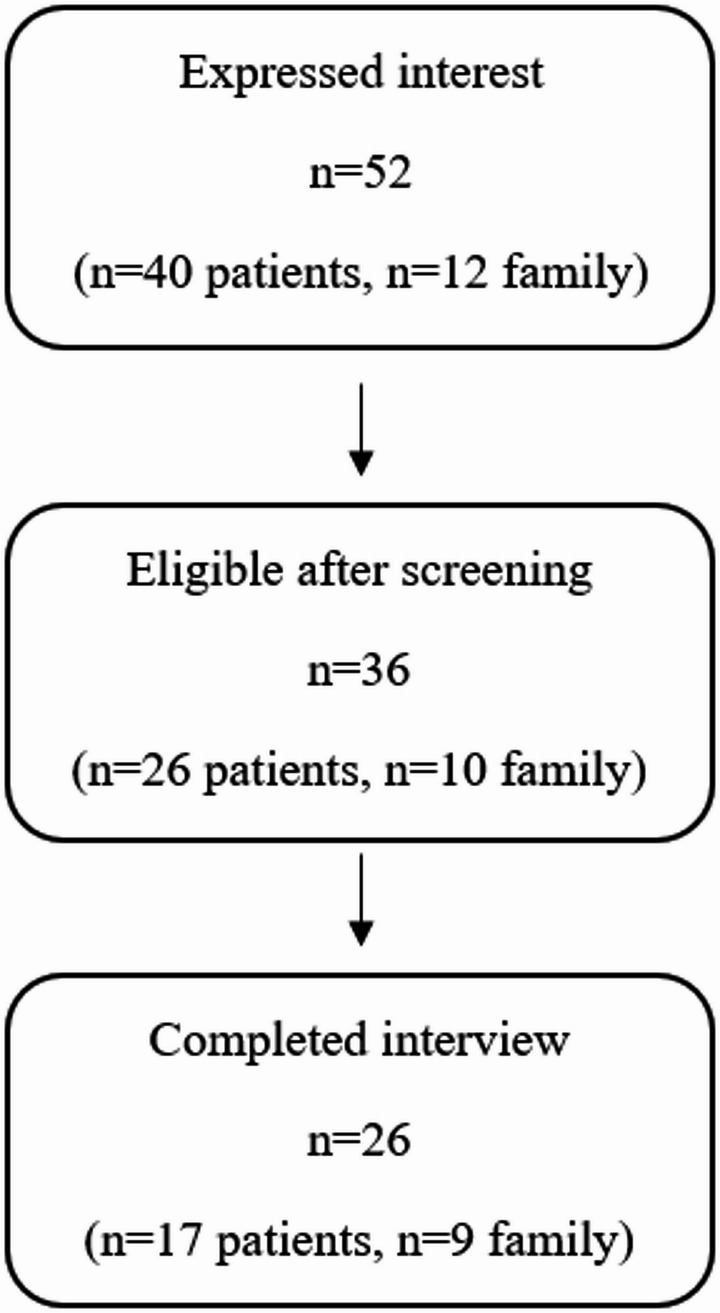
Summary of participant recruitment and completion

In total, 36 individuals were screened for inclusion and exclusion criteria (26 patients and 10 family members). Eligible individuals were offered an interview date and provided with a copy of the consent form for review. Ultimately, 17 patients and 9 family members attended and completed the interview. Participants who had scheduled interviews but did not attend were individuals who subsequently stopped responding to email or phone communication for unknown reasons. A visual summary of this recruitment pathway is presented in Fig. 1.

On the day of the interview, the interviewer (S. Carley) reviewed the consent with participants prior to beginning the interview questions. At this time, informed consent was obtained verbally or in writing, depending on the interview setting. Permission to audio-record the interview was also obtained. Before beginning the interview questions, participants were briefly informed about the purpose of the study and the motivations of the researchers and interviewer. No concerns were reported.

### Data collection

Two semi-structured interview guides (one for patients and one for family members) were developed for this study. The guides included 18 questions for patients and 17 for family members (See Supplementary Information, Appendix 1–2). Each question was accompanied by a set of potential probes, which were used flexibly depending on participants’ responses. The topic guide was informed by previous literature as well as clinical expertise (S. Adams & G.C. Adams) and was shaped to help address gaps in understanding specific to individuals with early psychosis and their families. As such, the interviews focused on experiences of psychotic illness, related risks, including the three examined risk factors, and how these were understood or addressed in recovery. All questions were open-ended to encourage an accurate depiction of the subjective experience of each participant, while minimizing the researcher’s influence.

Interviews were conducted either in person at an agreed upon location within the Royal University Hospital (where the EPIP program is located) or by phone, depending on participant preference and availability. The interviews were conducted by the first author (S. Carley), and no one else was present to ensure confidentiality. Interviews ranged from 30 to 90 min in length and were followed by a short debriefing to ensure participant comfort. No repeat interviews were conducted, as this was not required by the study design. Participants received a $40 cash honorarium for their participation. All interviews were audio-recorded and transcribed verbatim. All participants agreed to have their data be used in subsequent analyses.

### Data analysis

This study adopted a constructionist epistemological stance, meaning that “all knowledge is socially constructed” by in this case, the researchers [48] Because of this, the data was analyzed in an inductive (data-driven) manner. Thematic analysis was selected as the method of qualitative analysis, as it enables the identification of patterns or “themes” within a target population and supports nuanced interpretation. Braun and Clarke’s six-phase approach guided the analysis process [49].

The current study also follows a reflexive approach. Because codes and themes were developed inductively as the analysis progressed, and no pre-determined themes were outlined prior to coding, there was flexibility for the findings to evolve based on the researcher’s interpretations.

Transcripts were imported into NVivo 12 [50] and organized by participant group (patients and family members). The first author (S. Carley) read each transcript multiple times and listened to audio recordings to attend to tone, emotion, and nuance. Initial coding by S. Carley involved generating both semantic and early interpretive codes. Examples of early codes included “trauma linked to psychosis “mistrust,” and “important relationships.” This first round of coding was iterative and reflexive, accompanied by analytic notes documenting initial impressions and assumptions.

Coded segments were then collated into 13 broad initial groupings (six patient-focused and seven family-focused) to support early analytic organisation. Quotations were placed in more than one grouping when they addressed overlapping topics. Each grouping was reviewed in depth, and a second round of latent, interpretive coding was conducted by S. Carley to identify underlying meanings in participants’ accounts. Examples of these latent codes included “parents lacking understanding early in illness,” “sacrificing everything for cannabis,” and “rebirth of self through psychosis.” These codes were then clustered into 35 potential themes (19 from patient interviews and 16 from family interviews). Examples included “cannabis to cope with trauma,” “the restructuring of relationships/new normal,” and “the large toll on the family.”

Comparison across patient and family accounts revealed substantial conceptual overlap, and the analysis team agreed that combining these groups would produce a more coherent and holistic thematic structure. The 35 potential themes were refined into 10 preliminary themes representing shared meanings across participants. Example included “the importance of healthy attachments,” “the long and hard road to help,” and “inconsistent views and lack of universal understanding.” These preliminary themes, along with their supporting codes and quotations, were reviewed collaboratively with all co-authors throughout the analytic process. Interpretations were challenged, alternative perspectives were offered, and discrepancies were discussed until agreement was reached.

Further refinement was agreed upon and led by S. Carley, who consolidated the preliminary themes into five final themes by merging overlapping concepts, and removing themes with limited supporting data. Final theme names were developed collaboratively to reflect their central organising ideas. For reporting purposes, these five themes were then grouped into three overarching themes that depict the journey of psychosis from onset through recovery, as well as the understandings participants described across this process.

Rigour was supported through several strategies. Triangulation across participant groups was used early in the analysis to ensure that combining patient and family themes did not dilute unique perspectives. The first author also maintained a reflexive journal throughout the study, including immediately after interviews and during coding, to document evolving assumptions and analytic reflections. For example, during coding of transcript P20, a journal entry noted an initial pattern in how participants understood cannabis, and that there seemed to be a disconnect in understanding, which prompted further conceptual development. An audit trail was also maintained to document coding decisions, theme refinements, and the movement from codes to potential, preliminary, and final themes.

Transcripts were not returned to participants for comment or correction, nor did participants review the findings, in accordance with the study design and common practice in reflexive thematic analysis. All data were stored securely on DataStore, a password-protected institutional server accessible only to the research team.

### Reflexivity

As the first author, I conducted all interviews during my time as a graduate student and led most of the data analysis and writing. It is important to acknowledge that I am a white, Canadian, 27-year-old woman with academic privilege (BSc. Hon., MSc.), with personal prior experiences of mental health and relational adversity.

Accordingly, this background may bias how I interpret issues of trauma, attachment/ relational dynamics, and mental health recovery within participants’ accounts. However, I think these positionalities ultimately benefited me, as through this, I was able to engage with participants empathetically and attend closely to the nuances in their narratives. At the same time, I recognize that I have not experienced psychosis, early psychosis intervention, or substance use firsthand, and thus I will never fully understand the lived realities of those interviewed. This may have limited my ability to grasp certain subtleties.

Taken together, my positionality contributes both to the strengths as well as the constraints of the analytic process, which were addressed through ongoing reflexive engagement and collaborative theme review with all authors.

## Results

### Participant characteristics

Detailed characteristics of our sample are presented in (Table 1). Almost 65% of interviewed patients were male and almost 50% were unemployed. Also, 88% of patients were enrolled in the EPIP and 12% were attending SSS in Saskatoon at the time of their interview.

**Table 1:** Summary of demographic data for patients interviewed

Total interviewed	17
Clinic enrolled in at the time of the interview	EPIP: 15 SSS: 2
Gender	Male: 11 Female: 6
Occupation	Student: 3 Employed: 5 Unemployed: 8 Unknown: 1

Of the interviewed family members almost 90% were parents of the patients **(**Table 2).

**Table 2:** Summary of demographic data for family members interviewed

Total interviewed	9
Mother	5
Father	3
Sibling	1

### Thematic structure

**Fig. 2:**
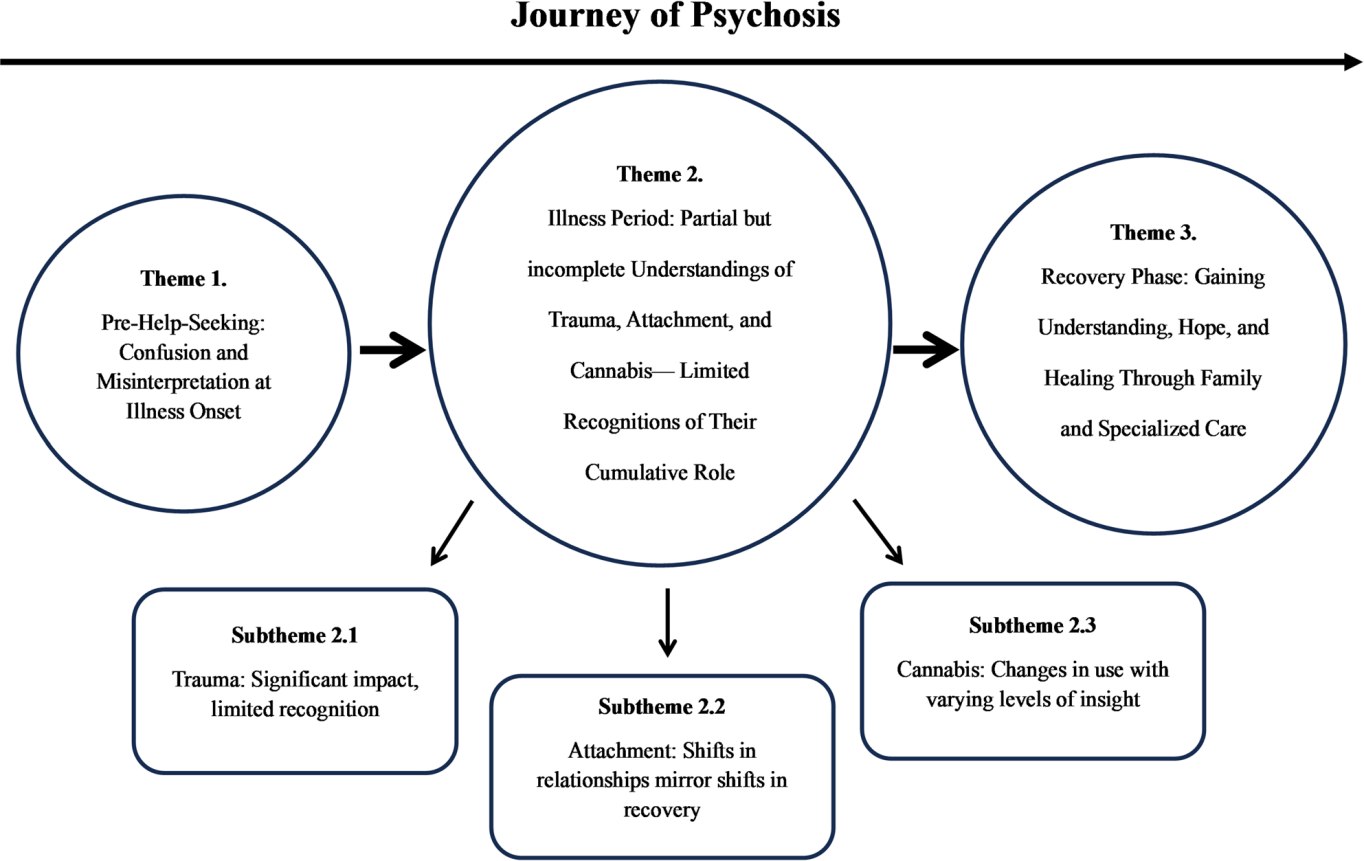
Thematic map of the three connected themes and subthemes across the psychosis journey

Three major themes and three subthemes were generated. A thematic map is shown in Fig. 2.

### Theme 1. Pre-help-seeking: confusion and misinterpretation at illness onset

The earliest stage of psychosis was characterized by limited understanding and frequent misinterpretation among both patients and family members. Rather than recognizing early changes as symptoms of psychosis, or as connected to underlying risks such as trauma, relationship strain, or cannabis use, participants commonly described confusion, uncertainty, and difficulty making sense of what was happening. Families often saw that something was wrong, but without a way to interpret these changes, they struggled to make sense of what those changes meant.

*F1: I was trying to put myself in his shoes.*,* but I couldn’t actually*,* I didn’t completely comprehend when he talked to me about these voices in his head…*.

*F3: I knew she needed help but I don’t know if I really identified it as psychosis*,* because I really didn’t know much about mental health at that time.*

Because of this, early signs were frequently dismissed or attributed to more familiar explanations such as normal developmental stages (being a teenager), personality changes, or poor choices (substance intoxication). At times, disrespectful behaviours were punished (e.g. removing the patient from home) or ignored (as just a phase) leading to responses that were misaligned with clinical needs.

*F5: I honestly think this is why psychosis is such a big problem*,* because first of all*,* I had never even heard of something like that*,* like I honestly wasn’t sure what that is. My mind never would have went there. I just thought*,* “ugh he is becoming disrespectful*,* he is rude*,* we should just kick him out” and like that was my mind set. I had no idea that he was like really slipping or already was in a psychosis. We just ignored him*,* we didn’t spend time with him…*.

This lack of early recognition often resulted in delayed help-seeking, with families engaging emergency services only when symptoms escalated. In some cases, this resulted in drastic interventions, including certified hospital admissions.

*F8: We tried to get her to go to sleep and she wasn’t going to sleep so it was*,* she was up all night. It was seven a.m. and I think she ran outside. And at that point we knew there was nothing we could do other than call ambulance*,* and we did.*

Patients themselves described even less awareness of their early experiences, often lacking the language or insight to articulate what was happening—even during the interviews. Many simply recalled the onset as “scary,” offering little elaboration. As a result, it was the family members that were able to better describe the overall confusion and lack of communication that characterised the onset of the illness, and for some, continues to persist.

*F6: To this day he will not say a word to me (regarding) anything has happened to him so I don’t know because he was gone for almost a year …So if anything happened to him*,* I don’t know because he won’t tell me.*

Unfortunately, the confusion surrounding psychosis was sometimes shared by health care providers, particularly when symptoms were unclear or fell within the prodromal phase. As a result, families often described feeling unsupported or dismissed during their early attempts to seek help, which reinforced their own uncertainty and frustration and further delayed an accurate understanding of what was happening.

*F3: … you take a person to the hospital five or six times and they just keep you there for 15 h and then send*,* send you home with no resources or anything*,* that’s*,* I mean that is a bit of a bone I have…*.

*F6: …I took him three times and they kept sending us home. One of them even said it sounds like a domestic dispute. And that was really frustrating.*

Once the diagnosis was finally made and a treatment plan was outlined, patients entered a slightly clearer (but still extremely challenging) stage of the illness, for which they often felt unprepared and (unfortunately) insufficiently supported. This further limited their understanding of what their symptoms meant and sometimes contributed to significant distress.

*P27: I was completely delusional for my hospital stay… When I was in the hospital for the 10 days*,* I should have been in there for another two weeks. They sent me home and from the day they sent me home to the six-week mark when I met my psychiatrist*,* were the absolute darkest days of my life.*

### Theme 2. Illness period: partial but incomplete understandings of trauma, attachment, and cannabis— limited recognitions of their cumulative role

Across accounts, participants attempted to make sense of trauma, attachment, and cannabis during the illness period. While many described these experiences vividly and with insight into specific aspects of their lives, their understanding of how these risks related to psychosis, and especially how they interacted cumulatively, was often partial, uneven, or varied between participants. It should also be noted that some participants varied in the extent to which they engaged with questions about the risks; meaning they did not always address certain topics, or provided minimal responses lacking depth (e.g., answering “no” without elaboration).

The following subthemes illustrate how participants recognized aspects of each risk yet rarely articulated how these vulnerabilities connected to each other or shaped their illness specifically.

#### Subtheme 2.1. Trauma: significant impact, limited recognition

Psychological trauma was seen as one of the most prominent risks across participants’ accounts. Most patients described one or more traumatic experiences (such as childhood abuse, sexual assault, or physical violence), yet their understanding of how their trauma related to their psychosis directly was often partial, uncertain, or indirect. While many participants recognized the emotional and relational consequences of trauma, few identified a direct connection between their trauma history and their diagnosis. Participants more often recognized how trauma related to relationships or substance use but had limited insight into how trauma alone—or cumulatively—specifically heightened psychosis risk.

Participants frequently described trauma as shaping their relationships and their sense of trust. One patient noted that unresolved trauma affected their ability to trust others, which created a scenario where their medication was not adequate.

*P23: I think that those trust bonds that…get destroyed with trauma… And medication only goes so far…*.

Others echoed this, describing long-term mistrust stemming from childhood abuse.

*P25: Yeah*,* like I have had childhood trauma…Not being able to trust people I guess is the main one.*

*P21: I think my trauma stems to the fact that I have trust issues because I still remember a lot of the abuse I got from my parents…*.

Family members also recognized this connection, explaining how past trauma shaped relational patterns that made their loved ones more vulnerable to harmful environments or influences.

*F8: The trauma that happened*,* and the rebellion*,* and the losing of her friends*,* and you know kind of pushed her further to people that weren’t good for her which pushed her further to being part of that party scene.*

Cannabis use was also closely intertwined with trauma, with several participants using cannabis to numb emotional pain or cope with distressing memories, suicidal thoughts, or loneliness.

*P11: …there’s some things that when you smoke weed you kind of forget about it…makes the pain go away…*.

*P11: I just started smoking to help…felt really lonely and isolated…thoughts of suicide at the time.*

*P22: It was actually after I was assaulted that I started using marijuana more.*

Although used in this regard, some participants did recognize that cannabis increased exposure to harm or worsened their emotional state, reflecting a partial awareness of the positive feedback loop that is created through increased trauma and cannabis.

*P5: …I started using cannabis even more… depression and suicidal thoughts kind of came at the same time while the violence grew…*.

Participants also briefly described how trauma contributed to worsening mental health and the emergence of psychotic symptoms.

*P11: Some of the stuff…when I was having the psychosis… were because of things that happened in the past*,* I think.*

*P21: …if you keep bottling it up… it just kind of… goes down to psychosis… And trauma has a lot to do with it.*

Though, this recognition varied widely, as several participants, including both patients and family members, remained uncertain about whether trauma played any role in their illness, reflecting limited or incomplete insight.

*P10: I’m not sure. I don’t know if trauma played a role (in my psychosis).*

*F5: I just felt like we were so little… I had no idea that the childhood abuse impacted him…*.

Participants also described increased vulnerability to severe outcomes such as suicidality, or exposure to harm, which may reflect a harmful result of leaving these risks unaddressed.

*P21: …Well I almost committed suicide when I was still a kid back then and I was just unhappy… with what life is…*.

*F8: …that is why we put her in the hospital because we were like okay this is getting too risky. Her life is getting worse*,* she might actually get into like harm*,* getting hurt unknowingly by just being at the wrong place at the wrong time.*

*P27: When I was a teenager*,* I was suicidal…that was when I was going through being molested…*.

One patient even acknowledged the severity of their experiences and expressed gratitude for having survived them.

*P22: When I was going through my episode*,* I am lucky I didn’t die honestly.*

#### Subtheme 2.2. Attachment: shifts in relationships mirror shifts in recovery

Across accounts, participants described a close connection between their relationships and their experiences of psychosis. Understanding of illness and recovery was often interpreted or supported through close relationships. For many, early relationship difficulties shaped how they understood their symptoms, and as recovery progressed, participants frequently described parallel shifts in their relationships, perhaps reflecting the role of others in their healing.

In early stages of illness, several patients described strained or troubled relationships within the family. Some traced this difficulty to longstanding relational trauma or abusive environments, which contributed to distrust, avoidance and isolation, and insecurity in later relationships.

*P24: … My only like female role models were ones that beat me and used me and called me stuff and so it was kind of hard to trust women like growing up and it kind of it was hard to interact and kind of be myself until my teenage years and then that kind of all went downhill after I moved because I was isolating myself again…*.

*P25: Well, I was isolating myself a lot…essentially, I couldn’t trust anybody…*

For others, relationship strain was intensified by more recent life events, such as trauma in adulthood or harmful behaviours such as substance use and illegal activity. Several participants identified cannabis use as a significant factor contributing to relational breakdowns, often leading to disconnection from family or feelings of being misunderstood.

*P26: I definitely stole from them*,* I took money I took like booze…I just wasn’t like a really good person when I was sick or when I was using cannabis… our relationship was strained quite seriously.*

*P10: I felt like my parents never understood me since I was a kid…and that was even more…intensified when I started using cannabis*,* because they don’t use.*

These narratives reflected core elements of insecure attachment (mistrust, emotional distance, and difficulty relying on others) [23, 24], which influenced how participants interpreted both their symptoms and the behaviours of people around them.

Even among patients with supportive relationships, the onset of psychosis often disrupted established patterns of connection. Confusion, fear, and changes in behaviour created emotional distance, leaving both patients and families uncertain about how to relate to one another and maintain closeness.

*F6: We used to be close before all of this happened and it just felt like… he wasn’t him for the past two years. Two*,* three years. But he is slowly getting back to himself but I know he will never fully be himself again.*

The emotional distance created by the illness also often left families struggling to understand their role in supporting recovery, particularly when their loved one was an adult. They expressed fear of crossing boundaries or appearing overly controlling, recognizing that too much involvement could be counterproductive.

*F8: We were very protective as parents not to not to “save” her in a sense*,* right? What 21-year-old wants to be saved. Nobody… it is like you think that you are being parented and then you just run faster and harder…*.

This uncertainty sometimes extended to cannabis use, where family members sometimes hesitated to set boundaries out of fear of further straining the relationship.

*F4: …Hearing her cough*,* you know*,* knowing that that must be some inhaling going on. This…is frustrating. We have been careful not to tell her that she couldn’t*,* or can’t do that in our house*,* because we are concerned of what that might cause.*

Patients also reflected on how their illness affected their understanding of their social world, noting difficulties in forming or maintaining relationships.

*P12: I realized I can’t really handle friends at same time of schizophrenia.*

However, as treatment progressed, many patients reported a gradual shift in perspectives. Insights gained through specialized programs, along with improved emotional stability, often made patients more open to receiving support, and more discerning about which relationships were helpful. Several described intentionally distancing themselves from unhealthy friendships while strengthening relationships that felt safe or supportive, resulting in a smaller social circle.

*P5: Oh yeah*,* I have lost tons of friends… Because I don’t smoke and I don’t drink that is all they do whenever they get together. So*,* I have a few friends now who don’t pressure me*,* who don’t judge*,* me who don’t do those things.*

*P22: I think I was in some unhealthy friendships before I went through my psychosis… and going through my psychosis just highlighted that*,* and so after that episode*,* I actually cut out quite a few people from my life…I didn’t make plans with family because I was with my friends…now that I’ve quit using cannabis I see my family a lot more.*

These renewed or strengthened relationships—whether with family, healthy friends, partners, or trustworthy clinicians, became paramount to successful treatment and recovery. Many understood the importance of their relationship during these difficult times, and emphasized how they contributed to them feeling supported, make healthier decisions, and maintain treatment engagement.

*P23: I think I was in a pretty safe environment in general which helped… I think that if I had been on my own maybe the psychosis would have gotten a lot worse or been a lot more out of control because I had my parents at least being there for me…*.

*P24: I give a lot of credit to my mom um for being there for me and kind of helping me move forward early on…now that I have moved out*,* she is always checking up on me and just making sure I am doing okay.*

*F2: Both me and my wife*,* his mother were supportive of him and whenever he goes*,* he comes with us now to go to the cottage and he brings his meds and we make sure he keeps that up to date…*.

Patients also described these relationships as serving as valuable models coping, supporting positive change and helping patients to replace previously engrained habits of drug use or other harmful coping methods.

*P11: I will talk to my girlfriend when feeling real stressed out*,* when I would normally take drugs.*

Strengthened connections also supported families’ own well-being, helping them cope with the challenges of caring for a loved one with psychosis, which in turn, enabled them to continue providing support throughout the recovery process.

*F3: I think that is our main coping strategies*,* is with each other.*

F4: I think *spouse’s name* and I are closer together,* closer now that we are now both retired and have more time… So*,* I think that probably allows us to support each other better and also to support (the patient) more.*

It should be noted, however, that there was one patient that still described a lack of connection to their family members, despite receiving treatment.

*P10: I just feel like they don’t know too much about my life… I don’t really have a relationship in a sense of like*,* what do you and your dad do…*.

#### Subtheme 2.3. Cannabis: changes in use with varying levels of insight

Previous cannabis use was self-reported as nearly universal among the patient sample, and most participants indicated that they continued to use it regularly up to the point of their enrolment in specialized programs. Although treatment engagement often led to reduced or discontinued cannabis use, patients’ understandings of its role in their mental health remained inconsistent and, at times, polarized.

Many described a gradual shift from viewing cannabis as helpful, to recognizing its harmful effects on relationships and mental well-being. Early on in their illness, cannabis was commonly understood as a positive force by patients, offering social connection and shared activities with peers.

*P5: Cannabis was socially acceptable*,* my friends all smoked. Yeah*,* it was (the) thing to do with your peers you know…*.

*P26: Cannabis was really like a social thing when I was a teenager. Like*,* all my friends used*,* and we kind of*,* I had a lot of friendships that kind of bonded over that.*

Many participants also initially perceived cannabis as helpful for managing anxiety, agitation, or distressing symptoms during the onset of illness, which reinforced the belief that cannabis provided relief rather than harm.

*P1: Well*,* cannabis is relaxing to me. You know it helps me relax and not be so edgy because of the voices… It helps me out. It keeps my mind off of it.*

*P23: Cannabis was definitely a way to manage those panic and anxiety attacks that I was having.*

However, as their illness progressed, several patients began to identify the negative consequences of cannabis use. This included worsening conflicts from disapproving family members, as well as increased withdrawal or isolation from supportive relationships.

*P11: …my parents were always super antidrug*,* so they weren’t too happy when they found out what I was using. That relationship got worse.*

*P18: Cannabis was ruining my relationships… I felt like I couldn’t talk to them (family and friends).*

A similar shift occurred with respect to mental health. While some participants initially believed cannabis reduced difficult mental health symptoms, many patients eventually recognized that it contributed to their anxiety, depression, cognitive impairment, or psychosis itself.

*P20: …I was smoking at the very beginning of my episode… I think it definitely made it worse.*

*P4: I feel that cannabis is portrayed as a heal all… I think that it creates or it can create some mental illnesses like psychosis… for some of us for sure…*.

For some individuals, recognitions of these harms led to discontinuation of cannabis use following treatment,

*P4: I haven’t used cannabis since I was in the hospital. So that is over two years ago.*

This often improved their overall well-being.

*P16: If I smoked now probably would get really bad anxiety and delusions… But now that I don’t smoke*,* I don’t get anxiety and delusions.*

Family members also demonstrated often more clear or consistent understandings of cannabis, including its association with psychosis severity, suicidality, or early vulnerability.

*F4: …he (the patient) told me that the reason that he did cannabis this time was because he thought it would kill him.*

*F5: I think… if you started cannabis early*,* and you do like too much of it*,* you are at a great risk of falling into psychosis.*

However, despite the intensive psychoeducation and treatment regarding cannabis that is received from specialized clinics such as EPIP, not all participants shared this growing recognition, and mixed opinions persisted during the interviews. For instance, some patients reduced their cannabis use, but did it for reasons unrelated to their illness, such as cost, suggesting that behavioural change may not always reflect a deeper awareness of risk.

*P10: I stopped using a large amount just because of a personal decision… I just decided it could be money… you don’t have money so you can’t go grab it today…*.

More concerning were the accounts of participants who continued to use cannabis despite ongoing psychotic symptoms, or who showed no shift in recognition and continued to view cannabis as beneficial for mood, socializing, or distraction.

*P21: I feel like cannabis doesn’t amplify psychosis that much. Like it gives you a good mental state and plus it allows you to socialize and stuff like that…*.

*P1: Cannabis actually helps me out a little bit. It like*,* draws my focus away from my illness*,* and then I don’t really pay attention too much to it.*

Family members’ perspectives also sometimes varied. While most clearly recognized the harms, others expressed limited awareness of cannabis-related risks and even described it as medicinal or harmless when used moderately.

*F2: With cannabis I imagine there are other drugs… Medicinal now too so I think’s it*,* not using it or you know*,* in moderation of course but…*.

### Theme 3. Recovery phase: gaining understanding, hope, and healing through family and specialized care

Participants described recovery not simply as symptom improvement, but also as a reconnection to supportive relationships, meaningful roles, and a renewed sense of self. While the specific processes of recovery were rarely named explicitly by participants, their accounts revealed several potential mechanisms through which specialized programs and relationships fostered understanding, stability, and hope. A sense of belonging, clear psychoeducation, and consistent emotional support appeared central to how patients and families made sense of the illness, regained confidence, and improved their optimism of the future.

For many, involvement in specialized early psychosis programs such as EPIP and SSS marked a turning point, both in their understanding of the illness, as well as in their broader recovery journey. Participants consistently expressed deep appreciation for the comprehensive, coordinated care they received, and repeatedly emphasized the depth of connection they formed with their health care provides. These close therapeutic relationships made patients feel genuinely cared for and valued, which helped counter the fear, stigma, and uncertainty that often followed their psychotic episode.

*P26: EPIP was amazing. I had support for everything. I am so fortunate that I got into that program because I know a lot of people probably struggle with that and mental health and psychosis*,* and they don’t get that chance to be a part of something like that.*

*P27: Honestly*,* I think the Early Psychosis Intervention Program really changed my life for the better after going through my psychosis. I feel like the nurses; my nurse was an angel…I am telling you she was an angel heaven sent. She is the kindest*,* sweetest*,* most caring yet supportive person I have ever met.*

*F7: I think the biggest thing is his nurse has been fabulous…. Like his nurse had been a life saver…we couldn’t have done it without him*,* he has been a life saver and has gone above and beyond.*

Psychoeducation and involvement of family members also played a central role. Families frequently described how learning about psychosis through the specialized clinics helped them better interpret their loved one’s behaviour. This can help in reducing confusion, increasing empathy, and giving families a clearer framework for understanding what their loved one was experiencing. This helps rebuild close relationships, and subsequently supports more effective involvement in recovery.

*F9: They have done a great job like explaining to us how psychosis works.*

In turn, the health care providers at the specialized clinics also listened to family members, which was meaningful.

*F8: The doctors at EPIP were good to us. They let us always come with them… And they listened. They were good listeners too.*

This added support for families is essential, as many family members described a strong sense of commitment to their loved one’s healing, which formed a stable emotional foundation during the most difficult stages.

*F9: He is my son*,* that’s that. I always put it into my mind that he is my son*,* I would not give him up.*

*F1: …Everything is always for him (the patient) in my life*,* it’s always the best for him*,* and he knows that…*.

Notably, one family member even demonstrated an ability to adopt their loved one’s perspective (a process known as mentalization) which may be crucial in building empathy and supporting communication, ultimately contributing to long-term treatment success.

*F1: I was trying to put myself in his shoes and try and think of different scenarios how I can better understand him…*.

As patients gained stability through treatment and support, many described a renewed sense of hope and future orientation. For some, recovery meant moving forward and rebuilding their identity by envisioning long-term goals and wishes.

*P5: I am fairly level headed now…. The only thing I wish was that I had a good career*,* well paying*,* family wife and kids. That is what I wish.*

*P14: I do want to go to university…*

Others described a valuable shift in meaning-making, reflecting on new perspectives that emerged from their illness, thus re-framing their negative experiences into a positive one.

*P27: I think my illness was a blessing in disguise because it really you know*,* woke me up in a sense and showed me that there is a different way about things.*

Patients also described a growing acceptance of their illness as part of making sense of their experiences. For instance, adjusting to long-term needs, such as ongoing medication, was often interpreted as part of a new, manageable way of living. This adjustment was often accompanied by a reduction in symptoms and an overall improvement in quality of life.

*P26: I think I am doing very well. I just got done work placement program. I am living on my own. I haven’t had symptoms in over a year. I am doing very well actually. I am still taking medication but that’s just kind of my life right now. I am keeping myself clean. I am keeping my space clean. I am really doing a lot better.*

## Discussion

### Summary of key findings

This study explored how individuals with first-episode psychosis and their family members understood psychosis and the roles of psychological trauma, insecure attachment, and cannabis use in both vulnerability and recovery. Three interconnected themes showcasing the journey of psychosis and the evolving understanding were identified: (1) *Pre-Help-Seeking: Confusion and Misinterpretation at Illness Onset; *(2) *Illness Period: Partial but Incomplete Understandings of Trauma*,* Attachment*,* and Cannabis—Limited Recognition of Their Cumulative Role*; and (3) *Recovery Phase: Gaining Understanding*,* Hope*,* and Healing Through Family and Specialized Care.*

Across all themes, participants described confusion about early symptoms, fragmented or partial understandings of risk factors, and gradual meaning-making supported through specialized treatment and supportive relationships. Importantly, these findings were drawn from individuals already engaged in specialized early psychosis services and considered clinically stable, suggesting that gaps in understanding may be even greater among those without access to such care.

### Findings in context

Consistent with prior literature [51], early symptoms of psychosis were widely misinterpreted. These misinterpretations strained family relationships and delayed help-seeking until crises occurred. As no participants provided statements contradicting this early confusion, the findings point to a broader lack of public knowledge about emerging psychosis. In publicly funded systems such as Canada’s, specialized services such as EPIP or SSS exist, but awareness and access remain limited due to factors such as self-stigma, lack of knowledge, or the absence of supportive relationships [52] Accessibility is further complicated by already strained health care systems. Although governments have recently invested significant resources into improving the quality of health care [53], one area that can be publicly targeted is the improvement of mental health literacy among families, schools, and even frontline health care providers. Enhancing this understanding could reduce the early confusion described by participants, and ease pressure on the health care system by enabling earlier, more structured interventions, rather than crisis responses.

Participants differed in their ability to articulate how trauma, relationships, and cannabis use related to their illness. Some appeared to develop deeper insight, potentially through specialized treatment, and implemented helpful changes such as quitting cannabis use. However, other participants’ understanding remained partial or conflicted. At times, some participants did not engage with questions at all, responding with brief “no” answers. This lack of engagement may reflect cognitive difficulties associated with psychosis. However, it may also indicate protective avoidance patterns related to an insecure attachment style, which could affect trust with the interviewer, increase symptomatology, or hinder communication [54] Alternatively, this may also reflect trauma-related avoidance [55, 56] rather than simple disinterest, potentially indicating unresolved contributing factors.

Supporting this interpretation, the first author (S. Carley) observed that participants who were less engaged during interviews were generally more difficult to converse with. We interpret this disengagement as potentially reflecting limited insight into these risks (and perhaps into their illness more broadly). Although these individuals were stable on treatment and self-reported improvements compared to intake, their disengagement and limited recognition of contributing factors may represent a meaningful distinction between a patient who is stable yet disconnected, and one who is stable while actively resuming normal life activities. At a broader level, the latter group may eventually return to work or school, resume healthy relationships, and might even experience a higher quality of life, which would reduce strain on the health care system and economy [2, 7, 8] This interpretation is offered by the authors; however, it is possible that the relationship operates in the reverse direction (i.e. the limited insight is due to a lack of engagement), and should be validated through future research.

**Fig. 3:**
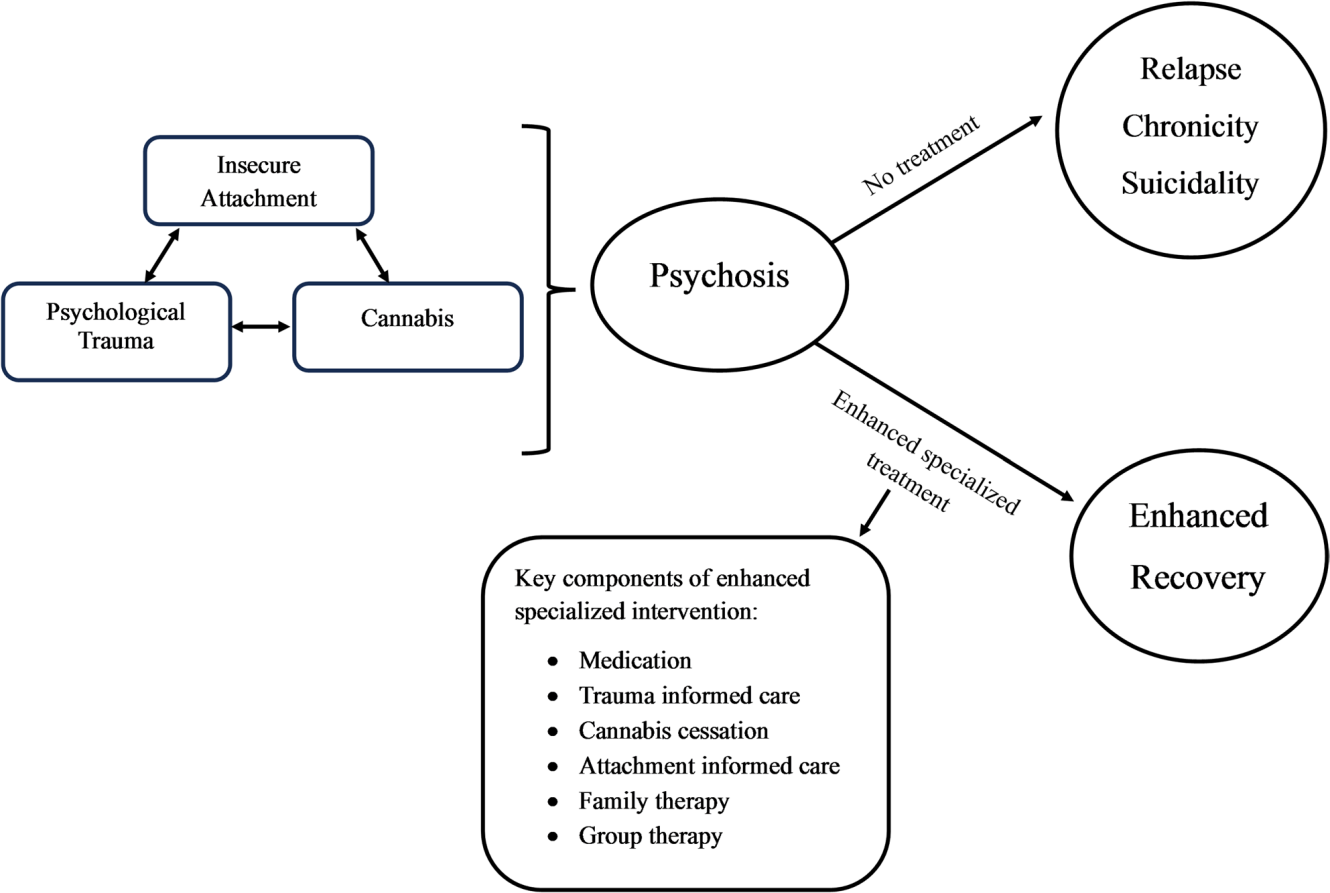
Potential risk pathway along with intervention targets for early psychosis. A visual model outlining the possible relationships among trauma, insecure attachment, and cannabis use as interconnected risk factors for psychosis, along with key therapeutic intervention points

A key contribution of this study is our interpretation of how trauma, insecure attachment, and cannabis use interact to create a cyclical pattern of vulnerability. From participants’ accounts, we infer that trauma often shaped mistrust in relationships and increased emotional avoidance, features of insecure attachment known to increase psychosis risk and delay help-seeking. Cannabis also frequently served as a means of belonging, which is consistent with research linking unmet attachment needs to substance use [57, 58] Over time, however, cannabis use added further difficulties, reinforcing maladaptive patterns, and creating a harmful feedback loop. Together, these risks compound into a cycle of risky behaviors, self-harm, substance misuse, relational breakdown, and trauma exacerbation. This cycle is typically interrupted only by intrusive interventions such as emergency services, hospitalization, or police involvement. This leaves individuals at a disadvantage, as they face sudden life changes alongside extensive treatment, making recovery even more challenging. To illustrate these relationships and potential therapeutic targets, we developed a theoretical model (Fig. 3**)** based on the thematic findings. This model emphasizes the cumulative—not independent—impact of these risks.

It should be noted that this model reflects our own analytic interpretation rather than participants’ conceptualizations. In fact, none of the participants recognized the full risk cycle, identifying only fragmented pathways, such as the connection between relationships and cannabis use. Some participants did not identify any connections, including concerning beliefs about the benefits of cannabis despite experiencing psychotic symptoms. These comments highlight not only the persistent gaps in understanding that remain even with specialized care but also reflect broader societal shifts in the attitudes and normalization of cannabis use, particularly in the Canadian context, perhaps due to recent legalization [59].

These findings underscore the need for integrated psychoeducation regarding these risks that move beyond simple identification and discussion, towards approaches that integrate these risks with one another, and within the context of their illness. Clinically, this could involve integrating novel approaches for addressing these risks, alongside regular medication. These approaches have been explored to some extent but remain limited in practice. Potential approaches could include: trauma-informed care [41] (which could help patients understand how their trauma interacts with their current relationships and substance use, as well as how leaving it unaddressed can be harmful); attachment-informed care [60] (which could help identify harmful relationships, repair secure ones, all while improving patients’ trust with clinicians); education on cannabis use [61] (which could support the shift from perceived early helpfulness to later cessation); family therapy [62] (which supports the recovery of healthy relationships, and equips caregivers with essential knowledge); and group therapy [63] (which supports patients lacking family involvement, and fosters belonging through developing connections with staff or peers).

Although these approaches have been explored in research and partially implemented in early intervention services such as EPIP and SSS, they remain far from standardized. Furthermore, accessibility barriers to early psychosis intervention services continue to exist, particularly for women and individuals experiencing socioeconomic deprivation [64, 65] This means that, while these services are helpful in theory, some patients with psychosis may never even access such clinics, which is an area that warrants further investment and research.

Recovery narratives in this study extended beyond symptom improvement to include reconnection with family, strengthened supportive relationships, and renewed future orientation. These experiences align with personal recovery frameworks emphasizing connectedness, hope, identity, and meaning-making [65,66] The centrality of relational support—reported by most patients and all family members—reinforces the value of attachment-informed and family-inclusive approaches [45, 67] In addition, early intervention programs themselves appeared to provide not only clinical stabilization but also relational consistency and belonging. This may be particularly valuable for individuals lacking supportive networks, as not all patients with severe mental illness, such as psychosis, have family members who are present or willing to engage as rigorously as the family members interviewed [68].

Despite substantial adversity, many participants described personal growth, greater self-awareness, and increased optimism. These patterns are consistent with literature on resilience and post-traumatic growth in psychosis [69], and suggest that early intervention programs should intentionally support meaning-making, identity reconstruction, and relational repair as core components of recovery.

### Strengths and limitations

This study uniquely contributes to the psychosis literature by integrating patient and family accounts to provide a holistic narrative of psychosis and by highlighting how individuals’ understandings evolve across the illness trajectory. Our study also proposes a theoretical model which illustrates how trauma, insecure attachment, and cannabis use may interact to amplify vulnerability. Additionally, the study demonstrates that even within specialized early intervention services, many individuals retain partial or conflicted understandings of these risks, highlighting clear opportunities to strengthen psychiatric care, psychoeducation, and relationally informed measures. The findings also indicate that improvements in mental health literacy are needed not only within clinical populations but also among the general public. Such efforts could help reduce early confusion, prevent emergency scenarios, enhance quality of life, and lower treatment costs.

Limitations include recruitment solely from specialized early psychosis intervention clinics. This may have produced a sample with higher treatment engagement and stability than typical first-episode populations and may also favored the view of these clinics. Because of this. individuals with more acute symptoms or limited service engagement may be underrepresented. The exclusive focus on trauma, attachment, and cannabis limited exploration of other potential risks (e.g., socioeconomic adversity, other substance use), which may reduce applicability to marginalized communities. Finally, data relied on self-reported narratives and did not receive clinical verification.

### Implications for policy, practice, and research

These findings highlight the need for improved early identification of psychosis, greater public and professional mental health literacy, and more integrated, holistic, and nuanced approaches to care. In fact, psychoeducation may be most effective when it addresses how trauma, attachment, and cannabis use interact, rather than treating them as discrete factors. In Canada, Clinical High Risk programs for psychosis may be strengthened by more explicitly and systematically assessing these factors in an integrated manner. Such considerations could be incorporated alongside commonly used structured assessments done in initial stages, such as the Structured Interview for Psychosis-Risk Syndromes (SIPS) [70]. Early, targeted intervention addressing these interrelated vulnerabilities may have important implications for these programs as these risks impact psychosis risk, development, symptom severity, and long-term outcomes.

Tailored approaches depending on the patient’s stage of recovery are also needed, which may help bridge gaps in insight and support sustained recovery. The variability in participants’ responses further support this and underscore the importance of tailoring psychoeducation to cognitive and emotional readiness rather than assuming uniform insight based solely on treatment duration. Importantly, psychoeducation efforts should not only convey clinical risks but should also explicitly address common misperceptions about cannabis that are shaped by public messaging and policy, to help patients and families navigate normalized or misleading societal narratives.

Finally, the strong role of family and trusted relationships suggests that early psychosis services should routinely incorporate family involvement and relationally based interventions. For individuals without stable networks, multidisciplinary teams may need to provide consistent relational support, whether through nurses or arranged external supports. Future research should examine how integrated trauma, attachment, and substance use models can be operationalized within early intervention services, and whether such approaches improve engagement, insight, and long-term outcomes.

## Conclusions

This study demonstrates that recovery from first-episode psychosis is shaped by the interplay of trauma, insecure attachment, and cannabis use across the illness trajectory. Nevertheless, gaps in understanding persist even within specialized care. These findings underscore the need to strengthen early psychosis services such as EPIP and SSS through holistic, relationally informed, and insight-tailored approaches. They also highlight the importance of improving public mental health literacy to promote earlier help-seeking. Future research should evaluate the proposed integrated risk model, examine its applicability in diverse and marginalized populations, and determine whether embedding such approaches into early psychosis treatment enhances engagement and supports long-term recovery outcomes.

## Supplementary information

Below is the link to the electronic supplementary material.

Supplementary Material 1

## Data Availability

The datasets generated and/or analysed during the current study are not publicly available due to confidentiality reasons, and the personal nature of the interviews, but are available from the corresponding author on reasonable request.

## Abbreviations

EPIP: Early Psychosis Intervention Program
SSS: Schizophrenia Society of Saskatchewan
SIPS: Structured Interview for Psychosis-Risk Syndromes
