# Supplementary material for: Exploring attachment, trauma, and cannabis use in psychotic disorders: a qualitative study of patient and family perspectives
Source: BMC Psychiatry. 2026 Mar 27;26:373. doi: 10.1186/s12888-026-08022-z (PMC13147706; doi:10.1186/s12888-026-08022-z)
Supplement: Supplementary file 1 — Supplementary Material 1 [file 12888_2026_8022_MOESM1_ESM.docx]

**Appendix 1. Interview guide for patients.**

**QUALITATIVE INTERVIEW FOR PEOPLE WITH LIVED EXPERIENCE**

**Welcome and introduction (items in quotes are suggested script)**

1. Interviewer introduces self and inquires how to address a participant: “We can be on a first name basis or use your preferred appellation”

2. Start by framing the interview: “You were selected to participate in this study because you have had experience with psychosis and cannabis use. We are going to ask you questions about your views with respect to a few important matters related to your experience related to this. The results will be used to better understand the risk factors that can create vulnerability for severe psychosis, and also to discover ways in which patients can be helped”.

3. The interviewer informs the participant that conversation will be audio recorded.

**Guidelines**

1. “I will ask you a series of questions. There are no right or wrong answers, only different points of view. Feel free to speak candidly about your experience and point of view.”

2. The interviewer may have to inform the participant that he/she is not able to share his/her thoughts on any subjects discussed. “My role as an interviewer will be to guide the conversation, not to provide my own experiences or thoughts.”

3. “The focus of this interview is to look at the possible link between cannabis and psychosis. We are going to ask you a few questions about your personal experience with these issues and your views with respect to a few important related matters.”

**Interview questions**

1. “I would like to ask some demographic questions to start the interview” *Interviewer asks name, age, sex at birth, gender identification, sexual orientation, ethnicity, occupation of interviewee.*

2. “Can you tell me a bit about your experience with psychosis?”

3. “What factors, if any, do you think might have led to your experience with psychosis?” *See what they say first before going into my risks.*

4. *Ask only if they do not talk about this in response to question 3.* “How would you describe your experience with cannabis?” *Let them answer.* “What benefits/downsides, if any, would you say it had?”

5. “How would you describe your cannabis use currently and over time?” *(The interviewer may need to provide temporal context to keep this item moving, such as when the participant start using? Are they using the same amount now as they were prior to diagnosis?)*

6. “Do you see any relationship between your use of cannabis use and psychosis?”

7. ““Do you have any history of trauma in your life? Can you describe how this trauma relates to your life experiences, or has impacted you, if at all?” *If they do not bring up psychosis/cannabis, probe with this after they answer.*

8. “Have you ever felt hopeless, unsafe or that your life was in danger?” *The interviewer might need to prompt the participant to give details about contributing factors.*

9. “How would you describe your living arrangements at the time of initial psychosis? Has it changed since then?” *If they do not mention cannabis/mental health being impacted, probe to see if it has relevance.*

10. “How would you describe your way of relating to others (friends, family, close people) over time (e.g. before and after cannabis use; before and after the diagnosis of psychosis was made*?)” The interviewer may need to prompt here as well. Is their social circle the same size? Do the participant go out more or less than they used to?*

11. “In what way(s) have these relationships impacted you?” *If they do not mention cannabis or psychosis, probe if they have impacted them in those areas.*

12. “How have these relationships changed over time, if at all?” *If they do not mention cannabis/psychosis impacting them, probe.*

13. “Are your family members involved in your treatment program?” *(Family member involvement is not always a positive experience; the interviewer should be prepared to receive any answers on the spectrum from extremely positive to extremely negative). If yes “*How have they been involved?’

14. “Have other members of your family ever struggled with mental health or substance abuse?” *As family members may not have consented to the sharing of their personal health information, try to steer this question more towards the relationship (e.g. mother, father, uncle, etc.) rather than names.*

15. “What impact, if any, did your gender or sexual orientation have on your experiences?”

16. “How would you evaluate the treatment you have been receiving?” *Let them answer,* “Have you ever been forced or coerced into treatment for anything?” *Probe for positives/negatives of treatment if they do not mention any.*

17. “What helped you cope with all the experiences/events that we discussed today”? *Ask only if not previously covered by the interviewee.*

18. “Is there anything else you think we should know, or did not discuss that you think is relevant?”

**Appendix 2. Interview guide for family members.**

**QUALITATIVE INTERVIEW FOR FAMILY MEMBERS WITH LIVED EXPERIENCE**

**Welcome and introduction (items in quotes are suggested script)**

1. Interviewer introduces self and inquires how to address the participant: “We can be on a first name basis or use your preferred name”
2. Start by framing the interview: “You were selected to participate in this study because you have been identified as the closest family member by one of our study participants who suffers from psychosis. We are going to ask you questions about your views with respect to a few important matters related to their diagnosis and related context. The results will be used to better understand the risk factors that can create vulnerability for severe psychosis, and also to discover ways in which patients can be helped”.
3. The interviewer informs the participant that the conversation will be audio recorded.

**Guidelines**

1. “I will ask you a series of questions. There are no right or wrong answers, only different points of view. Feel free to speak candidly about your experience and point of view.”
2. The interviewer may have to inform the participant that he/she is not able to share his/her thoughts on any subjects discussed. “My role as an interviewer will be to guide the conversation, not to provide my own experiences or thoughts.”

**Interview questions**

1. “I would first like to ask some demographic questions to start the interview.” Interviewer asks name, age, occupation of interviewee and relationship to patient.
2. “Can you tell me how you’ve come to understand your loved one’s psychotic episode(s)?” *Wait to see what they say first – then probe if needed. Want to get a sense of what their loved one is like. Probes: “Can you give me a sense of the timeline before the episode?” “What did you observe prior to the episode?” “What might have caused the episode?”*
3. “How would you describe your loved one’s journey with cannabis use?” *See what they say first, then could ask about first time use, frequency, amount, etc.*
4. “How have you felt over time about your loved one using cannabis (in general/in your presence/your home)?”
5. “To what extent, if at all, are you and your loved one’s views on cannabis and psychosis in agreement?”
6. “I am wondering about whether your relationship with your loved one has been affected by cannabis use, and if it has, how so?” *Let them answer*. “What about their psychosis, how has that affected your relationship, if at all?”
7. “I am wondering about the presence of any trauma history in your loved one’s life?” *See if they naturally connect the trauma to cannabis use/psychosis, if not go to question 8*
8. “I am wondering if you see any connection between your loved one’s cannabis use and trauma? How do you view this connection?” *Let them answer.* “How do you see the connections, if any, between their psychosis and trauma?”
9. “How would you describe your loved one’s way of relating to others over time? Have you seen any changes?” *The interviewer may need to prompt with e.g. before and after cannabis use; before and after the diagnosis of psychosis was made.*
10. “What connections, if any, would you describe between their close relationships and cannabis use? *Let them answer.* “What about any connections if any to psychosis?” *If yes.* “Can you tell me a little more about that?”
11. “Do you see a connection between your loved one’s living arrangements and their cannabis use?” *Let them answer.* “What about connections to their mental health?”
12. “I’m wondering whether you’ve ever felt if your loved one was at risk for self harm or suicide?” *Let them answer how they understood what led up to that before asking probes. Probing questions: “What do you think might precipitate that?” “Can you tell me about when that occurred?” (If they say they attempted suicide).*
13. “How involved are you in your family member’s treatment program (EPIP) and what do you think of it?”
14. “What has your experience of treatment with your loved one been like?” *Probe if it they had to resort to drastic measures if they do not mention.*
15. “How would you evaluate the treatment that your loved one has received? *Let them answer.* If necessary: “What has been good? What has been not good?”
16. “I am wondering whether other members of your families have ever struggled with mental health or substance abuse?” *The interviewer reassures the participant that they do not need to answer any questions in the interview that they feel uncomfortable with if they hesitate to answer this question.*
17. “Is there anything else you would like to share with us, or anything we did not cover that you think is relevant?”
